# Event and Comment

**Published:** 1911-05-15

**Authors:** 


### DENTAL REGISTER.

**Vol. LXV. May 15, 1911. No. 5**

### EVENT AND COMMENT.

REORGANIZATION OF THE NATIONAL ASSOCIATION. At the meeting in Denver last year a scheme was adopted for calling a convention sometime during the year to consider a plan of reorganization for presentation at the meeting in Cleveland this year. The Cleveland meeting is only two months away and so far as we know no convention has been held or even been called, and we shall probably either have another postponement or be called upon to adopt some hastily prepared report of the committee, prepared on the train or at hurried conferences between sessions. Now, why in the name of common sense, this kind of inaction is tolerated by earnest and sensible men who are vitally interested in our profession's welfare we cannot understand. The editor of the *Western Dental Journal* in the April issue says that the Reorganization Committee, will submit a report for adoption at Cleveland, but before doing so it desires an expression of opinion from every State Dental Association as to whether they will become constituent bodies of the National Association. Now what an absurdity that is, would any sane man join a church on the promise that by and by it would have a suitable creed for him to subscribe to? State Societies are not going to be satisfied with a promise, they must know what is to be expected of them; and we don't believe many will want to subscribe to a constitution which they have had no voice in making. There will be no time to fully discuss this matter at Cleveland unless the convention could meet two or three days earlier than the National Association. There may be some good reason for the delay in this matter that we are unacquainted with, but we are getting tired of such dilatory practices and feel that we must say something. So far as professional progress is concerned there is no more important problem before the annual meeting, and as it is the "Kings business," it not only requires haste, but the most careful consideration our wisest councilors can give to it. Dr. Root in his editorial says a letter will be sent to all the State Associations asking them to vote on the question of becoming constituent members, does he not know that many of the State Societies have already held their annual meetings? How does he expect to get a reply from such organizations now?

THE DENTAL EDUCATIONAL COUNCIL OF AMERICA. A joint meeting of the Executive Committee and the Committee on Colleges of this Council was held in Chicago April 9, 1911. One of the subjects up for discussion was how best to stimulate a sympathetic interest in the profession in behalf of the work which this body is now endeavoring to do.

It is believed that the aim and object of this organization is not very well understood by the profession, and the Secretary was instructed to invite the co-operation of the editors of all the dental journals with the hope that a more general professional interest might be secured.

Doubtless all are aware that there has been a great deal of general criticism of American dental colleges. Justice compels us to say that after two years of work of this Committee, we are free to confess that there is some justification for some of the complaints which have been made. By reading our circular you will note that this Council is composed of five representatives from each of the three national bodies, and therefore it should be regarded as representative. Our function is purely advisory, and we are endeavoring in a systematic way, to find out what the real cause of complaint may be. By making a thorough investigation of the kind of dental education now being offered we hope, in due time, to make recommendations based upon our findings. A report for this year's work will be made at the next annual meeting of the three national bodies, and we hope that our organization will be continued for at least another year.

The question of how reforms in dental education can be enforced very naturally arises. What is the use of an inquiry if suggestions intended to reform are made by a fair minded commission if they cannot be enforced?

We are frank to admit that an inquiry, no matter how searching, which may be conducted by this or any other commission, will not necessarily provide men with a conscience. In other words, it is not an easy matter to make even educators honest unless they want to be. There are ways and means, however, which we believe can be adopted with good results. The one legal body that we know of that can enforce the law, is the State Board of Dental Examiners, and at the proper time, should occasion demand this, we believe that proper action can be procured.

There is, however, another side to this question. Many of the differences that have arisen between the Colleges, State Boards and the profession at large, are due to prejudices which find their origin in a lack of mutual confidence and respect. That there are dental colleges which are not worthy of the name, every thoughtful and well-informed member of our profession will admit. On the other hand, most of our dental colleges are doing good, honest work, and this fact must not be lost sight of.

During the past year we have heard some talk of the advisability of inviting the Carnegie Foundation to investigate our dental schools. We have no fault to find with such an investigation, and would cheerfully co-operate with any properly constituted commission, in an endeavor to unify or raise the standards of dental education. Some time has now elapsed since the Carnegie Foundation made its report on medical education. Just how much good its report has done, we do not know. The question to which we would like to invite your thoughtful consideration is,— Are fairminded men representing our three great national bodies who are familiar with the needs of dental education, and who are making a study of its problems, entitled to the co-operation and assistance of the profession at large? If the State Boards, Dental Colleges and their Faculties, and our State Dental Socities will get behind this movement, and the information of how this can be done is given them through the medium of our Dental Journals, it is easy to prophecy that the results obtained will make for the general up-lift of dentistry. The best results, however, cannot be obtained in one or two years, and we believe that it is only by patient, persistent and honest effort that permanent improvements can be hoped for.

I shall be glad to hear from you, and can promise in advance, that I will frankly, fairly, and to the best of my ability answer any questions you may care to ask respecting the work of this Council and its organization. If you are interested, will you please write an editorial along broad educational lines to be published in your May or June Number?

With sentiments of esteem, and kind personal regards, I am

Sincerely yours,
HENRY E. BANZHAF,
Secretary.

The above letter comes to us both officially and personally with an invitation of such serious importance that we can not refuse to make some comment, although the matter is so weighty that a longer and more detailed study is required than we can now give to it. There can be no question but a very considerable improvement in dental instruction is necessary to equip future practitioners to meet the ever increasing responsibilities of our profession. The average practitioner who is conscientious about the services he is called upon to render knows that he falls far short of doing his whole duty to his patients, and, moreover, he sees the work of many college products that is so inferior as to make him believe that the dental colleges are not doing their duty to the profession or public either. If he could look at the matter from the standpoint of the college he would find that the teaching matter of today is more than double that of twenty years ago when he passed through the college course. He would also find that there has been not more than twenty-five per cent. increase in entrance standards or teaching time. It is true the teaching equipment has very materially increased and in many schools it is all that could be asked; but there has been little or no increase in permanent remuneration that would justify professional men in hazarding business success in practice to prepare themselves for teaching as a business. We don't believe that the managers of any considerable number of our teaching institutions are willfully or intentionally unmindful of the highest professional interests, or that they are conducting their schools for money only. We have the good fortune to know personally most of the men in charge of the colleges in America and have had many opportunities of conferring with them on pedagogical as well as business relations and we have become thoroughly convinced that were they free from the financial necessities they would to a man come out strongly for ideal standards.

If, therefore, our premise is true there can be little doubt as to the form of remedy. We must have to begin with educated instructors who are not only capable, but are filled with self sacrifice and zeal for the work; then we must have proper and well equipped buildings for their use: and lastly we must have suitable materials upon which they can work to the best advantage. If we could have these three factors ideally developed our problem would solve itself.

Now friends Pruyn, Summa, Banzhaf and others, if I were asked to make your report for you, I should certainly incorporate the above as a statement of facts to be worked out by such practicable resources as may be available. The demand for high grade dental services is greater today than ever before and the supply is entirely inadequate for the needs of the people, and unless some material increase in our equipment is speedily provided the professional increment of the future will degenerate. Your plan of considering this subject as a National issue is excellent, as it can so be discussed without personal prejudice or conflicting and selfish interests. Can not all the National Medical and Dental organizations combine in a propaganda for the foundation of completely endowed and equipped Medical and Dental schools at such strategic points as to give us a National system of professional education? Millions of money is being contributed every year for less humane and up-lifting causes than the great healing art, which sooner or later is of vital interest to every man. If we could formulate a national scheme of professional education there can be no possible doubt that it would make successful appeal to the wealthy philanthropy of our country. In some places the state institutions can be strengthened and utilized and in the other sections endowed institutions could take over the existing private schools until we could have a complete system covering the entire country which would establish the standard, if it could not control the business of professional education. It could be made so strong that all private teaching would have to level up or suffer in rank. If your Council is organized for a permanent result set your aim on a worthy ideal and stand strongly for it.

